# Leakage Current Mechanism of InN-Based Metal-Insulator-Semiconductor Structures with Al_2_O_3_ as Dielectric Layers

**DOI:** 10.1186/s11671-016-1232-0

**Published:** 2016-01-13

**Authors:** X. Wang, G. Z. Zhang, Y. Xu, X. W. Gan, C. Chen, Z. Wang, Y. Wang, J. L. Wang, T. Wang, H. Wu, C. Liu

**Affiliations:** Key Laboratory of Artificial Micro- and Nano-structures of Ministry of Education, School of Physics and Technology, Wuhan University, Wuhan, 430072 People’s Republic of China

**Keywords:** InN, Al_2_O_3_, MIS

## Abstract

InN-based metal-insulator-semiconductor (MIS) structures were prepared with Al_2_O_3_ as the gate oxides. Surface morphologies of InN films are improved with increasing Mg doping concentrations. At high frequencies, the measured capacitance densities deviate from the real ones with turning frequencies inversely proportional to series resistances. An ultralow leakage current density of 1.35 × 10^−9^ A/cm^2^ at 1 V is obtained. Fowler-Nordheim tunneling is the main mechanism of the leakage current at high fields, while Schottky emission dominates at low fields. Capacitance densities shift with different biases, indicating that the InN-based MIS structures can serve as potential candidates for MIS field-effect transistors.

## Background

III-Nitrides, with excellent optic and electronic properties, can be widely used for solar cells, optical wave guides, high-speed electronics, and terahertz emitters [1]. Among them, InN has the lowest effective mass of electrons and the highest mobility, and thus, it is a promising semiconductor for applications in high-speed electronics such as field-effect transistors (FETs) and high-electron-mobility transistors (HEMTs). One of the major obstacles that limit the performance and reliability of these transistors for high-power radio-frequency (rf) applications is the high gate leakage [2]. To solve this problem, structures like metal-insulator-semiconductor (MIS) and metal-oxide-semiconductor (MOS) have been developed by using SiO_2_ and Al_2_O_3_ as the dielectric layers [3, 4]. However, none of MIS or MOS structures has been reported on InN electronic devices yet.

In recent years, high-quality InN films have been grown by molecular beam epitaxy (MBE) [5, 6]. Although the surface electron accumulation is not completely explained and solved, p-type carriers have been confirmed in Mg-doped InN by indirect evidences such as measurements of electrolyte-based capacitance-voltage (ECV) [7], temperature-dependent Hall effect [8], thermopower [9], and photoconductivity [10]. All efforts mentioned above lay a good foundation for the fabrication of high-quality Mg-doped InN MISFETs and MIS-HEMTs.

The Al_2_O_3_ dielectric layer has been widely used in MIS and MOS structures due to its relatively larger dielectric constant compared to that of SiO_2_. Atom layer deposition (ALD) has many advantages in growing the Al_2_O_3_ dielectric layer such as low temperatures and pinhole-free growth. Hence p-type InN-based MOS and MIS structures with Al_2_O_3_ as the dielectric layers are promising to be applied for FETs, HEMTs, and other kinds of thin-film transistors (TFTs).

In this work, Mg-doped InN films were grown on c-plane sapphire with GaN buffer layers by MBE. Al_2_O_3_ dielectric layers were then grown by ALD. Top Cr/Au electrodes were made by thermal evaporation, while bottom electrodes were welded In dots. Surface morphology of InN films was improved with increasing Mg doping concentrations. An ultralow leakage current density of 1.35 × 10^−9^ A/cm^2^ at 1 V was obtained. The leakage mechanism, capacitance density versus frequency (*C*-*F*), and capacitance density versus voltage (*C*-*V*) of this InN-based MIS structure were also investigated.

## Methods

Mg-doped InN films were grown on c-plane sapphire with GaN buffer layers by using radio-frequency plasma-assisted molecular beam epitaxy (rf-MBE, SVTA 35-V-2). Thin GaN buffer layers, with a thickness of 50 nm, were grown under the optimized conditions with the substrate temperature at 760 °C and Ga source temperature at 1020 °C on c-plane sapphire [11, 12]. InN films were then grown for 2 h under the optimized conditions reported previously, i.e., setting the In source temperature at 770 °C, substrate temperature at 450 °C, and N flow rate at 2.65 sccm [11, 12]. Mg doping in InN films was performed with Mg source temperatures at 300, 310, 320, 330, 335, and 340 °C, respectively. Al_2_O_3_ dielectric films, with a thickness of 50 nm, were prepared with a growth rate of 0.1 nm/cycle by ALD (Beneq TFS-200) by using the precursors of trimethyl aluminum (TMA) and H_2_O. The detailed growth conditions can be found in our previous work [13–15]. Cr/Au (15-nm Cr and 50-nm Au) were fabricated on Al_2_O_3_ layers by thermal evaporation with templates of 150 × 150 μm^2^ in area as the top electrodes. In dots were welded on InN layers as the bottom electrodes. InN films were examined by high-resolution x-ray diffraction (HRXRD, Bede D1) and atomic force microscopy (AFM, SPM-9500J3, Shimadzu). The *C*-*F*, *C*-*V*, and leakage current density versus voltage (*I*-*V*) characteristics were measured by using a semiconductor device analyzer (Keithley 4200, Keithley Instruments).

## Results and Discussion

**Fig. 1:**
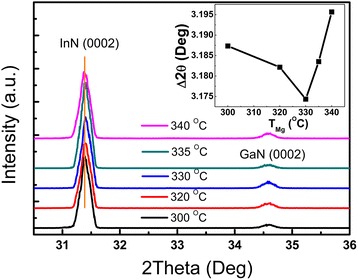
XRD spectra of InN films with different Mg source temperatures from 300 to 340 °C. The *inset* shows the corresponding differences of peak centers between InN and GaN versus Mg source temperatures

Figure 1 shows XRD patterns of InN thin films grown on sapphire substrates with 50-nm GaN buffer layers. *Y*-axis offsets were added to make XRD spectra clearly presented from each other. No peaks around 33° were found, excluding the existence of indium droplets on the InN surface. (0002) InN and (0002) GaN peaks were located around 31.4° and 34.6°, respectively. With the increase of Mg source temperature, the diffraction peaks of (0002) InN films shifted, while those of (0002) GaN were preserved. The full width at half maximum (FWHM) of (0002) InN films with GaN buffer layers was about 0.27° in contrast to 0.42° without GaN buffer layers. This proved that the quality of InN films was significantly improved with GaN buffer layers as reported previously [16]. To analyze the influence of Mg doping in InN films, InN and GaN peaks were fitted with a Gauss model to get more accurate peak information. The inset showed Mg source temperatures versus differences of peak centers between InN and GaN. The difference decreased as Mg source temperature increased from 300 to 330 °C, while it increased with higher Mg source temperature. Taking Bragg’s law 2*d*_*hkl*_sin*θ* = *nλ* and hexagonal interplanar distance formula *d*_*hkl*_ = 1/((4(*h*^2^ + *k*^2^ + *hk*)/(3*a*^2^) + *l*^2^/*c*^2^))^1/2^ into consideration, the crystal parameter *c* of Mg-doped InN films firstly decreased and then increased as Mg source temperature increased. This could be explained by the Mg doping position changing from the interstitial sites to the substitutional In-sites, forming In_1-*x*_Mg_*x*_N alloys as the Mg source temperature increased. This result is in agreement with Wang’s results that with the increase of the Mg cell temperature, InN was firstly n type being slightly Mg doped, then became p type with enough Mg acceptors higher than ionized donors, and finally became n type again because of over-doped Mg [7–10].

**Fig. 2:**
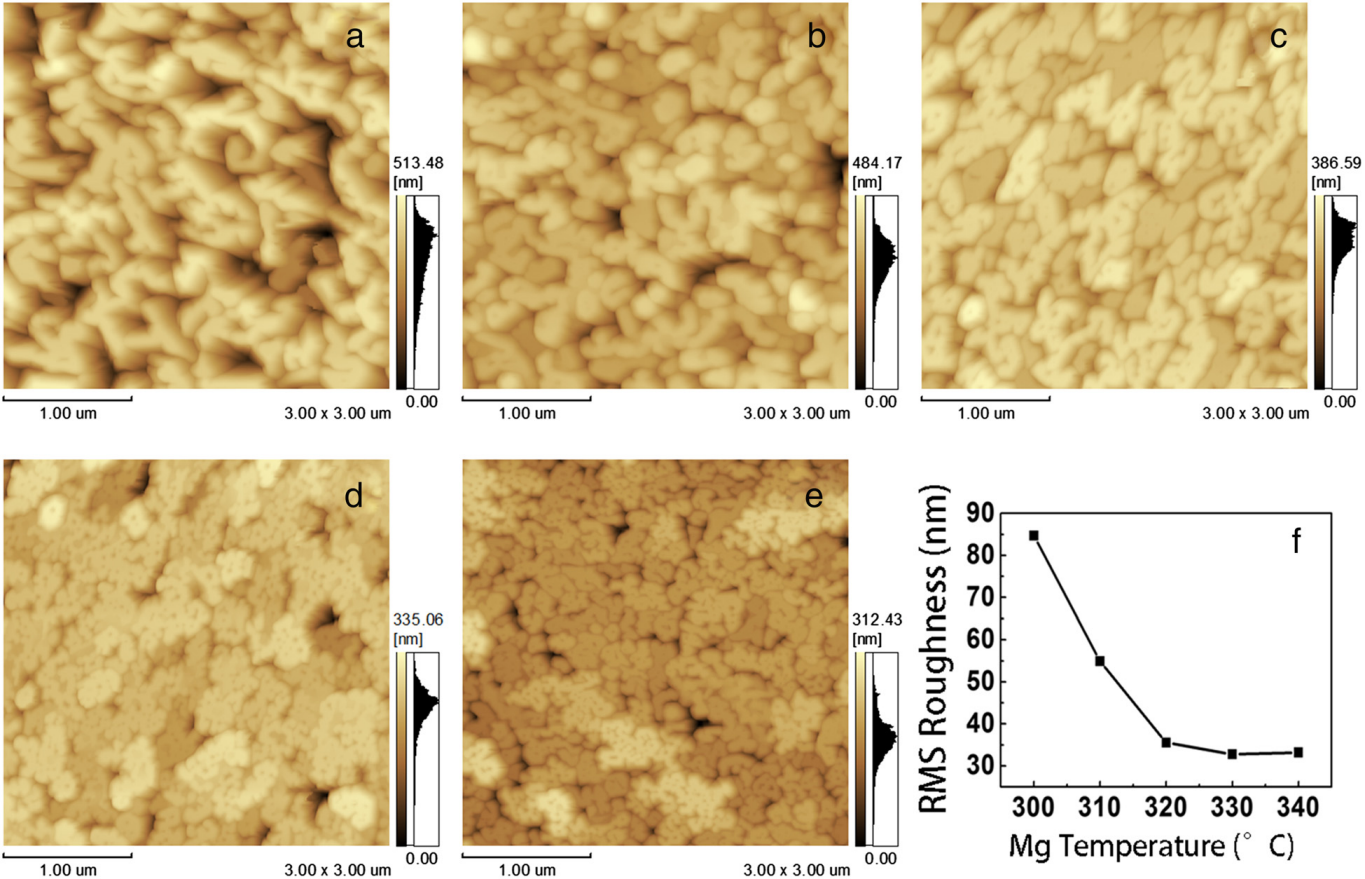
Surface morphologies of InN films doped with Mg at different source temperatures: **a** 300 °C, **b** 310 °C, **c** 320 °C, **d** 330 °C, and **e** 340 °C. **f** Root-mean-square (RMS) roughness versus Mg source temperatures

Figure 2 shows the AFM morphologies of InN films in an area of 3 × 3 μm^2^. The best morphology of Mg-doped InN films was achieved at 330 °C with the lowest root-mean-square (RMS) roughness of 32.8 nm. As seen from Fig. 2a–e, the grain sizes decreased as the Mg source temperature increased. Meanwhile, the RMS roughness decreased from 84.7 to 32.8 nm as the Mg source temperature increased from 300 to 330 °C, indicating that the InN surface became smoother with increasing Mg doping concentrations. This may be caused by introducing more In_1-*x*_Mg_*x*_N alloys as catalysts. Such a positive result provides more potential for InN to be applied in MIS and MOS structures.

**Fig. 3:**
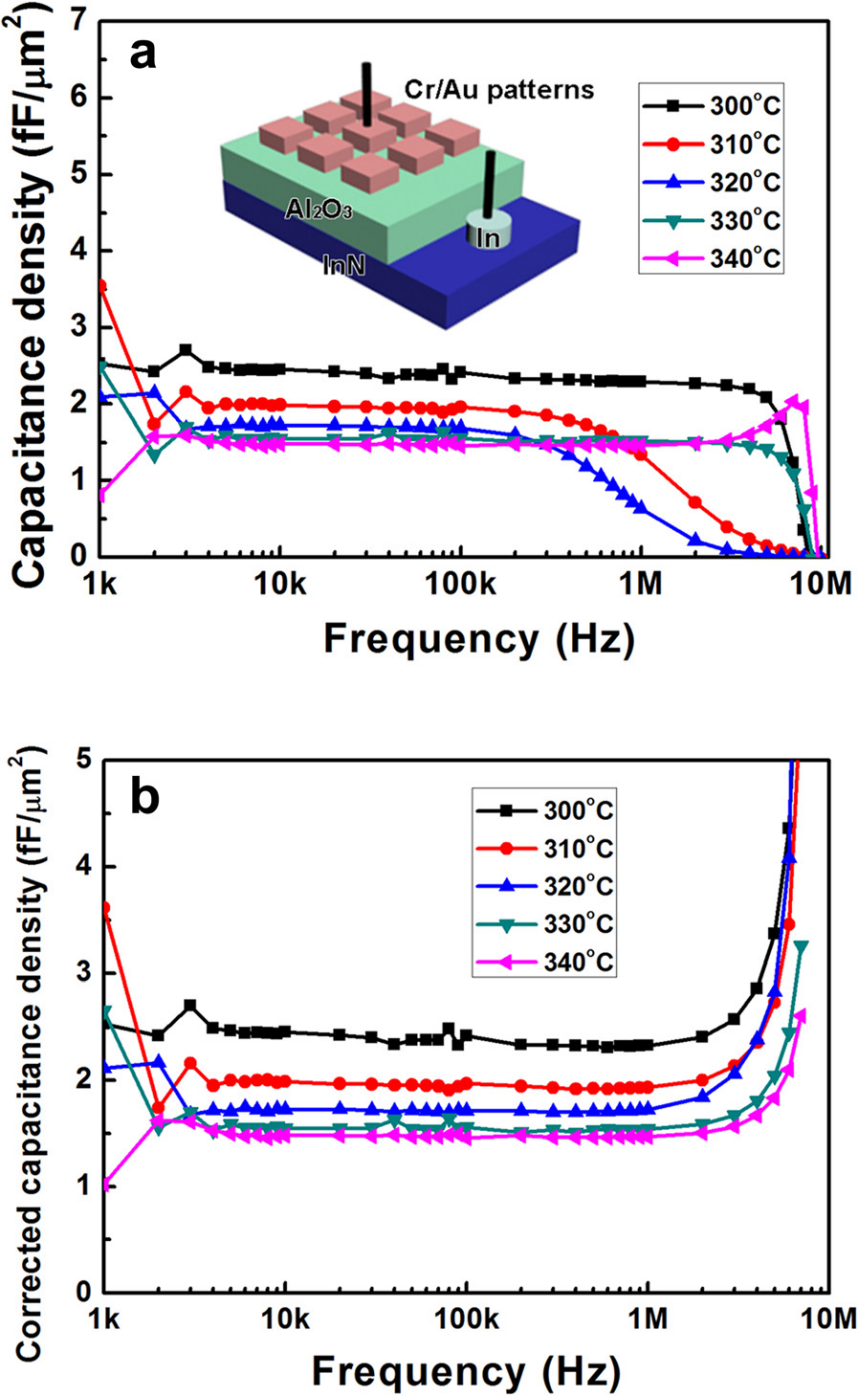
**a** Measured and **b** corrected *C*-*F* characteristics of InN-based MIS structures with different Mg source temperatures. The *inset* shows the schematic diagram of an InN-based MIS structure

Figure 3 shows the *C*-*F* characteristics of the InN MIS structure with no bias. Capacitance density is an index depending on the frequency because the capacitance *C* = (*ε*_r_*S*)/(4π*kd*) and the dielectric constant *ε*_r_ are related to the frequency. In Fig. 3a, taking the InN MIS structure with Mg source temperature at 320 °C into consideration, capacitance densities are about 1.8 fF/μm^2^ from 5 to 100 kHz, corresponding to a dielectric constant of 10.2. For the frequencies lower than 5 kHz, capacitance densities were hard to obtain because of low conductance. For the frequencies from 5 to 100 kHz, the capacitance densities almost remained constant. In addition, the capacitance densities decreased with increasing Mg source temperatures. Furthermore, for the frequency higher than 100 kHz, the capacitance densities deviated from the true ones, which were attributed to the relative resistances of Mg-doped InN films. The sheet resistances of Mg-doped InN films were 633, 2392, 31844, 525, 359, and 134 Ω for different Mg source temperatures set at 300, 310, 320, 330, 335, and 340 °C, respectively. Considering the MIS structure consisting of a junction capacitance *C*, a junction conductance *G*, and a series resistance *r*, the true capacitance *C*_t_ could be corrected by measured capacitance *C*_m_, measured conductance *G*_m_, and frequency *f* as follows: *C*_t_ = *C*_m_[1 + (*G*_m_/*ωC*_m_)^2^], with *ω* = 2*πf* [17]. Hence, the measured capacitances deviated from the true ones at high junction conductance. Defining the quality factor *Q* for a parallel circuit by *Q* = (*ωC*_m_)/*G*_m_, the true capacitance should be measured for *Q* ≥ 5 [17]. The transitions of *Q* from larger than 5 to smaller than 5 happened at 2 MHz, 300 kHz, 200 kHz, 2 MHz, and 4 MHz for Mg source temperatures at 300, 310, 320, 330, and 340 °C, respectively, which matched the relative resistances and the deviation frequencies in Fig. 3a. This shows that the measured capacitance densities deviate from the real ones with turning frequencies inversely proportional to series resistances. Furthermore, Fig. 3b shows the corrected capacitance densities. The deviations at high frequency from 100 kHz to 2 MHz were corrected, while capacitance densities at frequencies higher than 2 MHz were not so reliable because Q << 5.

**Fig. 4:**
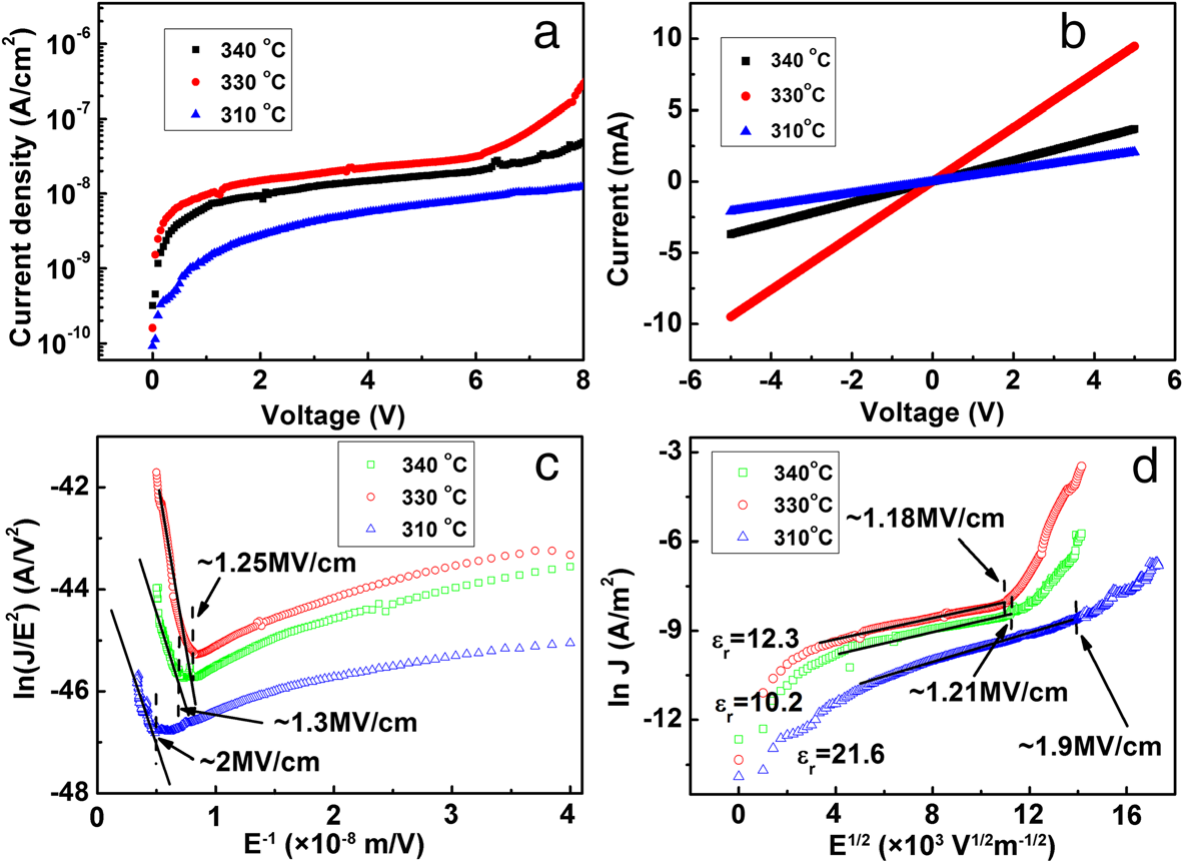
**a** *I*-*V* characteristics of InN-based MIS structures with different Mg source temperatures. **b** Ohmic behavior between InN films and In electrodes. **c** F-N tunneling as well as **d** Schottky emission plots of the leakage currents versus different Mg source temperatures

Figure 4a shows the *I*-*V* characteristics with the bias from 0 to 8 V. The ohmic contacts between InN films and In electrodes are shown in Fig. 4b. An ultralow leakage current density of 1.35 × 10^−9^ A/cm^2^ at 1 V was obtained for the sample with Mg source temperature at 310 °C, leading to little dielectric loss at low frequencies. Therefore, the Al_2_O_3_ films demonstrate excellent insulating properties and passivation abilities on InN films for both MISFET and MIS-HEMT systems.

The leakage mechanism is investigated by using models of Schottky, Fowler-Nordheim (F-N), and Frenkel-Poole (F-P) tunneling emissions. Figure 4c shows the relationship between ln(*J*/*E*^2^) and the reciprocal of electric field (*E*^−1^). When the field was above 1.3 MV/cm for Mg source temperature at 340 °C, above 1.25 MV/cm for that at 330 °C, or above 2 MV/cm for that at 310 °C, a linear relationship was observed, which meant that the conduction mechanism was governed by F-N tunneling at high fields. At low fields, ln(*J*) versus *E*^1/2^ was also linear in Fig. 4d, meaning that the conduction was governed by Schottky emission. As Mg source temperature decreased, the conduction mechanism changed at fields of 1.21 MV/cm for 340 °C, 1.18 MV/cm for 330 °C, and 1.9 MV/cm for 310 °C, which also proved that the leakage mechanism of the Al_2_O_3_/InN structure followed the F-N tunneling mechanism at high fields and the Schottky emission mechanism at low fields. The fitted relative dielectric constants were 10.2, 12.3, and 21.6 for different Mg concentrations in InN films. Furthermore, the Frenkel-Poole (F-P) emission model was applied to analyze the leakage mechanism of the Al_2_O_3_/InN structure (not shown). No linear relationship between ln(*J*/*E*) versus *E*^1/2^ was found. Hence, it can be concluded that the leakage mechanism of the Al_2_O_3_/InN structure follows the F-N tunneling when the field is above 1.2 MV/cm and the Schottky emission when the field is lower than 1.2 MV/cm.

**Fig. 5:**
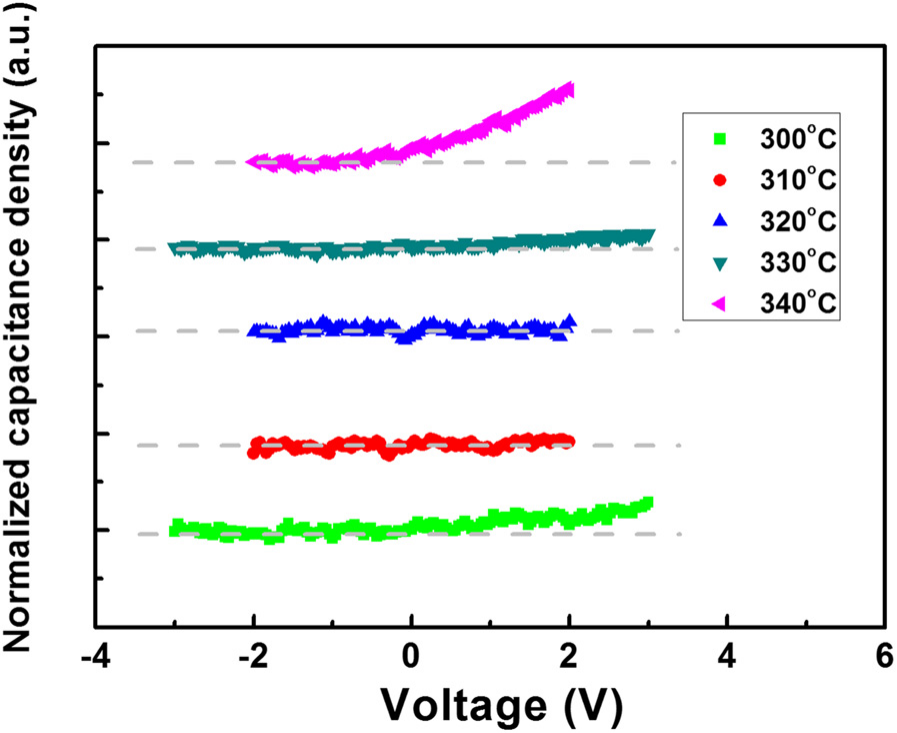
Normalized *C*-*V* characteristics of InN-based MIS structures at 1 MHz with different Mg source temperatures

Figure 5 shows the normalized *C*-*V* characteristics of the InN MIS structures at 1 MHz. The capacitance densities at 1 MHz with no bias deviated from the true values, which is the same with the *C*-*F* results. When the gate voltage was applied, the capacitance densities shifted because of the induced charges at the interface between InN and Al_2_O_3_. Normalization was adopted to make these shifts more clearly visible by (*C*-*C*_0_)/*C*_0_ with *C*_0_ being the capacitance density at 0 V. *Y*-axis offsets were also added. When a positive bias was applied, an increased capacitance was measured due to more induced electrons at the interface, and thus, the surface state was n type. It was observed from Fig. 5 that the curves of the capacitance densities versus voltage varied with Mg source temperatures. Significant shifts occurred at 300, 330, and 340 °C, while none was found at 310 and 320 °C. This could be caused by Mg acceptor and ionized donor recombination around 320 °C, indexing a transition from n to p type, and then from p to n type again with increasing Mg dopants. Such shifts indicate that the Mg-doped InN MIS structures can be used for MISFETs.

## Conclusions

In conclusion, InN-based MIS structures were fabricated with high-quality Al_2_O_3_ thin films as dielectrics. An ultralow leakage current density of 1.35 × 10^−9^ A/cm^2^ at 1 V was achieved. At high frequencies, the measured capacitance densities deviated from the real ones with turning frequencies inversely proportional to series resistances. It can be concluded that Fowler-Nordheim tunneling is the main mechanism of the leakage current at high fields, while Schottky emission dominates at low fields. The Mg-doped InN MIS structures can serve as potential candidates for MISFETs.
